# Nanotechnology Enables Novel Modalities for Neuromodulation

**DOI:** 10.1002/adma.202103208

**Published:** 2021-10-19

**Authors:** Xiao Yang, Eve McGlynn, Rupam Das, Sergiu P. Paşca, Bianxiao Cui, Hadi Heidari

**Affiliations:** ^1^ Department of Psychiatry and Behavioral Sciences Stanford University Stanford CA 94305 USA; ^2^ Stanford Brain Organogenesis Wu Tsai Neurosciences Institute Stanford University Stanford CA 94305 USA; ^3^ Wu Tsai Neurosciences Institute Stanford University Stanford CA 94305 USA; ^4^ Department of Chemistry Stanford University Stanford CA 94305 USA; ^5^ Microelectronics Lab (meLAB) James Watt School of Engineering University of Glasgow Glasgow G12 8QQ UK

**Keywords:** nanomaterials, nanotechnology, neural interfaces, neuromodulation

## Abstract

Neuromodulation is of great importance both as a fundamental neuroscience research tool for analyzing and understanding the brain function, and as a therapeutic avenue for treating brain disorders. Here, an overview of conceptual and technical progress in developing neuromodulation strategies is provided, and it is suggested that recent advances in nanotechnology are enabling novel neuromodulation modalities with less invasiveness, improved biointerfaces, deeper penetration, and higher spatiotemporal precision. The use of nanotechnology and the employment of versatile nanomaterials and nanoscale devices with tailored physical properties have led to considerable research progress. To conclude, an outlook discussing current challenges and future directions for next‐generation neuromodulation modalities is presented.

## Graphical abstract

Recent advances in developing novel nanoscale neuromodulation strategies with less invasiveness, improved biointerfaces, deeper penetration, and higher spatiotemporal precision are discussed. The employment of versatile nanomaterials and nanoscale devices with tailored physical properties has led to considerable research progress in nanotechnology‐enabled electrical, optical, chemical, acoustic, and magnetic neuromodulation modalities, and cross‐modal neuromodulation strategies using nanomaterials as energy transducers.

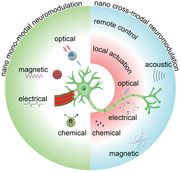

## 1 Introduction

Neuromodulation is the process of changing neural activity by delivering electrical, optical, chemical, acoustic or magnetic stimuli to targeted neural tissue.^[^ ^1^ ^]^ It provides powerful tools both for understanding the brain function, and for modulating the activity of malfunctioning neural circuits to ameliorate disease progression.^[^ ^2^ ^]^ The use of neuromodulation in neuroscience research has enabled a wealth of discoveries on functional connectivity in neural circuits.^[^ ^3^, ^4^, ^5^, ^6^, ^7^, ^8^ ^]^ Moreover, neuromodulation strategies capable of improving, restoring and substituting motor, sensory and cognitive functions have led to therapeutic avenues and prosthetics for treating neuropsychiatric disorders. Achieving minimally invasive neuromodulation on specific cell types and neural circuits in deep brain regions with high spatiotemporal resolution is the ultimate goal for neuromodulation,^[^ ^2^ ^]^ although it has yet to be achieved with current neuromodulation technologies.

Here, we focus on how emerging nanotechnology is galvanizing novel neuromodulation strategies, with an emphasis on recent research progress on nanotechnology‐enabled neuromodulation modalities with less invasive surgical procedures, improved bio‐implant interfaces, deeper brain accessibility, and higher spatiotemporal resolution. We discuss how nanotechnology is enabling specific neuromodulation modalities, such as electrical, optical, chemical, acoustic and magnetic, as well as grafted forms of cross‐modal neuromodulation strategies using nanomaterials as energy transducers. In the end, we provide an outlook on future endeavors in advancing neuromodulation strategies for fundamental research and clinical translation.

## 2 Roadmap for Neuromodulation Strategies: From Macroscale to Nanoscale

**Figure 1:**
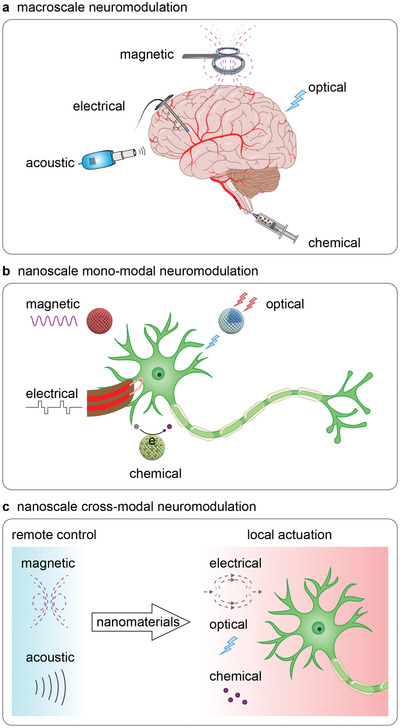
From macroscale to nanoscale neuromodulation. a) Classic electrical, optical, chemical, acoustic, and magnetic neuromodulation at the macroscale. b) Nanotechnology‐enabled new neuromodulation at the nanoscale. c) Nanomaterial‐mediated cross‐modal neuromodulation.

Modern neuromodulation tools have a history spanning six decades,^[^ ^1^ ^]^ ranging from classical approaches including deep brain stimulation (DBS), to recent approaches including optogenetics and chemogenetics.^[^ ^9^ ^]^ Neuromodulation strategies based on electrical stimulation are not limited to DBS (**Figure** **1**a), but also include sacral nerve stimulation and spinal cord stimulation.

The well‐established electrical neuromodulation approaches have been used to interrogate impaired neural circuits in a broad range of neurological disorders, and were approved for clinical treatments since the 1990s.^[^ ^9^ ^]^ Notably, DBS of the subthalamic nucleus (STN) has been used to treat and improve long‐term motor functions in Parkinson's disease patients.^[^ ^10^ ^]^ Although effective to help alleviate the symptoms of a wide range of diseases and disorders, DBS requires the invasive placement of electrodes, and they may fail after implantation following tissue responses.

In addition to electrical approaches, recent development in alternative neuromodulation approaches include optical, chemical, acoustic and magnetic modalities. Optogenetics^[^ ^11^, ^12^ ^]^ has rapidly become a powerful neuromodulation tool which revolutionized the interrogation of specific cell types and neural circuits with high temporal resolution (Figure 1a). The key advantage of optogenetics over electrical approaches is its capability for cell type‐specific interrogation. While electrical stimulation lacks specificity, optogenetics can control the function of defined events in specific cell populations by combining genetic and optical manipulations. Although optogenetic applications are limited by light attenuation in neural tissue^[^ ^13^ ^]^ and the need for implantation of optical fibers, recent reports of red‐shifted opsins that can be used without intracranial surgery hold great promise for noninvasive implant‐free deep brain transcranial optogenetics.^[^ ^14^ ^]^

With similarly high cell‐type specificity, chemogenetics represents another important neuromodulation modality (Figure 1a). Combining genetic and chemical manipulations, chemogenetics utilizes engineered receptors and exogenous molecules specifically targeting those receptors (e.g., G protein‐coupled receptors (GPCRs) and clozapine *N*‐oxide for designer receptors exclusively activated by designer drugs (DREADD)^[^ ^4^ ^]^) to control cell activity. A key advantage of chemogenetics over optogenetics is its noninvasive activation/inhibition as it does not require the implantation of optical fibers, and thus it has been widely used to evaluate and establish causality between neural circuits and behavior. However, as chemogenetics relies on the diffusion of drugs throughout the body, its onset time on the scale of minutes is slower than that of optogenetics on the scale of milliseconds.

In the other realms of neuromodulation, acoustic,^[^ ^5^, ^15^ ^]^ and magnetic^[^ ^16^ ^]^ stimulation approaches are important neuromodulation modalities due to their noninvasive nature. Because sound waves and magnetic fields can penetrate deep into tissues, they can be used to directly activate or inhibit neurons in deep brain regions. With deep penetrating and spatial focusing capabilities, focused ultrasound (FUS) has been demonstrated to transcranially deliver acoustic energy to modulate neural activity in humans.^[^ ^6^ ^]^ Moreover, in recent years interest has risen significantly in the application of sonogenetics, the acoustic counterpart of optogenetics and chemogenetics. Most recent in vivo studies demonstrated sonogenetic modulation of target neurons expressing an ultrasound‐responsive protein,^[^ ^17^ ^]^ although further investigations are still needed to characterize modulated behavioral alterations.

In the regime of magnetic neuromodulation, transcranial magnetic stimulation (TMS; Figure 1a) is the most commonly used magnetic stimulation method for therapeutic applications, primarily for the treatment of drug‐resistant depression.^[^ ^18^, ^19^ ^]^ However, with a typical penetration depth of a couple of centimeters,^[^ ^20^ ^]^ TMS is only applicable to stimulate neurons that lie close to the outer layer of the human brain and is generally limited by its spatiotemporal precision and lack of target selectivity.

In recent years, active research efforts have been revolutionizing neuromodulation modalities through emerging nanotechnology (Figure 1b).^[^ ^21^ ^]^ On the one hand, the versatile nanoscience toolkit pushed neuromodulation approaches that have long been associated with bulky devices toward miniaturized devices with soft mechanics, densely packed components and sustained performance. These nanoscale tools with improved spatial resolution and localized targeting capability allow for seamless integration with the neural tissue.^[^ ^22^ ^]^ On the other hand, nanomaterials with favorable physical and chemical properties distinct from their bulk counterpart hold promise for overcoming some of the limitations of classic neuromodulation strategies at the macroscale.

Recognizing the intrinsic merits and limitations of individual modalities, a variety of nanotechnology‐enabled grafted neuromodulation modalities that take advantages of the strengths while sidestepping the limitations of individual modalities have emerged in recent years (Figure 1c). Nanomaterials and nanoscale devices allow the efficient energy transduction from one modality that excels in deep brain penetration, to another modality that excels in localized neuromodulation with a high spatiotemporal resolution, thereby enabling new grafted forms of neuromodulation modalities.

## 3 Nanotechnology‐Enabled Monomodal Neuromodulation

Compared to their bulk counterpart, materials and devices with nanoscale dimensions can circumvent some of the aforementioned limitations of current neuromodulation approaches by enabling minimally invasive and seamlessly integrated neural interfaces for localized neuromodulation with high spatiotemporal resolution.

First, miniaturized materials and devices exhibit structural and mechanical properties closer to the cell populations in the central nervous system (CNS), thereby enabling more biocompatible and less invasive neural interfaces. Since bending stiffness scales with the material's Young's modulus and the cube of the thickness,^[^ ^23^ ^]^ research aimed at improving mechanical properties has focused on using more flexible materials^[^ ^24^, ^25^ ^]^ and/or optimizing the device geometry.^[^ ^23^ ^]^ When their feature sizes reach nanoscale, materials become flexible and compliant and thus are able to seamlessly integrate with neural tissue with negligible foreign body response.^[^ ^23^ ^]^ Second, materials and devices at the nanoscale enable localized neuromodulation with high spatial precision at targeted brain region with reduced off‐target side effects. Nanoscale building blocks allow for dense packing and concentration in a small volume, thereby improving the spatial resolution and controllability of neuromodulation.^[^ ^22^ ^]^ Third, one fascinating characteristic of nanomaterials is that they start to exhibit new physical and chemical properties distinct from their bulk counterpart when scaled down to the nanoscale regime, which can be harnessed to enable novel neuromodulation modalities. The dependence of nanomaterial characteristics on the size, shape, and surface properties can be employed to fine‐tune nanomaterials with tailored properties for innovative neuromodulation modes.

Here, we discuss emerging modalities of neuromodulation that are enabled by nanotechnology. Neuromodulation strategies based on implantable or injectable materials and devices at miniaturized scales enable new forms of DBS, optogenetics and many other neuromodulation modalities with improved capabilities.

### 3.1 Electrical Neuromodulation

**Figure 2:**
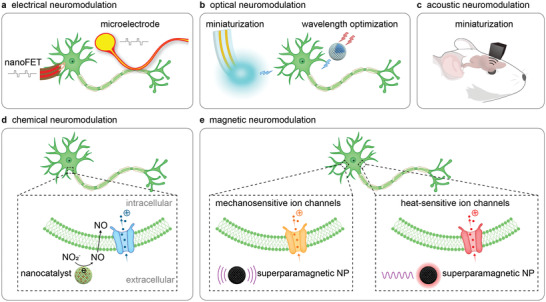
Nanotechnology‐enabled modulation. a) Electrical neuromodulation of neurons using microelectrode and nanoFET array. b) Optical neuromodulation of neurons using miniaturized light source and UCNP. c) Acoustic neuromodulation of the brain using miniaturized ultrasonic neural stimulator. d) Chemical neuromodulation of neurons through locally generated modulatory molecule. e) Magnetic neuromodulation of neurons using superparamagnetic nanoparticles. For illustrative purposes, diagrams are not necessarily drawn to scale.

Multiple lines of evidence suggest that structural and mechanical distinctions between electrical devices and their neuron targets can lead to disruption of the native tissue that precludes the devices from stably interrogating and modulating neural activity over time.^[^ ^23^, ^26^, ^27^, ^28^ ^]^ To minimize these disparities, flexible electronics with nanoscale building blocks have been actively investigated to deliver electrical stimuli to the CNS for electrical modulation (**Figure** **2**a). A representative example of device geometry optimization is the ultraflexible neuron‐like electronics with open mesh structure, which shows a stable gliosis‐free neural interface capable of chronic recording^[^ ^23^ ^]^ and stimulation^[^ ^29^ ^]^ (Figure 2a, right). On the other hand, mechanical properties of implanted devices can also be improved by using more flexible materials. For example, soft neural implants with elastic properties similar to neural tissue were fabricated and implemented as electronic dura mater^[^ ^24^ ^]^ and auditory brainstem implants^[^ ^30^ ^]^ for sustained electrical stimulation in the spinal cord or the cochlear nuclei, respectively. Notably, spatiotemporally targeted neuromodulation of spinal cord motor neurons has been shown in individuals with spinal cord injury to restore voluntary control over previously paralyzed muscles and improve neurological recovery.^[^ ^8^ ^]^ The mechanical compliance of these miniaturized electronics allows them to adhere conformally on the curved surfaces in the CNS, and highlights their applications in precision neuromodulation and neuroprosthetics in a way not possible for conventional DBS electrodes. Flexible electronics with multi‐contact electrodes could provide higher‐resolution electrical readout and better control of the electric field for next‐generation DBS in clinical settings.^[^ ^10^ ^]^ However, comprehensive dosage testing on stimulation parameters is needed to establish safe stimulation protocols.

Scaling down to the nanometer regime, nanowire field‐effect transistor (nanoFET) arrays and complementary metal–oxide–semiconductor (CMOS) nanoelectrode arrays represent two active interfacing technologies for stimulating neuronal cultures in vitro. NanoFET arrays were demonstrated to achieve spatially resolved extracellular modulation of neuronal activity through integrated junctions with the axons and dendrites of live mammalian neurons,^[^ ^31^ ^]^ and to record multiplexed intracellular action potential in primary neurons with full amplitude^[^ ^32^ ^]^ (Figure 2a, left). Compared to a stimulating electrode which is often larger than 100 µm in diameter, a nanoFET junction has an area of 0.02 µm^2^ which mimics the dimension of a synapse. Nanofabrication allows for custom configuration of nanoFET device array geometries, with spacing between individually addressable nanoFETs down to 100 nm. Using 0.1 nA current stimulation, recognizable action potential spikes were elicited in single neurons.^[^ ^31^ ^]^ In the intracellular regime, CMOS nanoelectrode arrays are capable of intracellular stimulation to activate ion channels of neurons across a large network of cultured neurons.^[^ ^33^ ^]^ As a step forward, human pluripotent stem cell (hPSC)‐derived brain region‐specific organoids and assembloids^[^ ^34^, ^35^ ^]^ can serve as a platform to investigate how these electronics with nanoscale building blocks modulate neural development and to potentially test therapeutic approaches for neuropsychiatric disorders.

### 3.2 Optical Neuromodulation

The use of optogenetics for neuromodulation was generally limited in terms of light penetration, invasiveness and the need for genetic modifications. Recently, significant progress has been made by the power of nanotechnology to address each of these limitations.

The limited light penetration in the visible spectrum and invasiveness of conventional tethered optogenetics can be addressed or improved by miniaturizing device footprint or tuning wavelength to the near‐infrared (NIR) window. In 2013, a wirelessly powered optogenetics system with a microscale light‐emitting diode (µ‐LED) was built and injected into the mouse brain.^[^ ^36^ ^]^ This optoelectronic system consists of active semiconductor and metal materials bonded to a mechanically compliant epoxy support. The size of the µ‐LED (Figure 2b, left) is approaching that of a single neuron, and is at least 10 times smaller than conventional tethered optical fibers. Employing µ‐LEDs in a self‐contained stimulation system allows animal subjects to move freely instead of being limited by the fixed location of the light source. The µ‐LED systems are ultralight and battery‐free, offering unique opportunities for behavior and social interaction studies on freely moving animals in complex environments.

Another nanotechnology strategy to address the limitations of light penetration and invasiveness of conventional optogenetics is via injectable upconversion nanoparticles (UCNPs)^[^ ^37^ ^]^ (Figure 2b, right). Since the scattering and absorption of light by biological tissue are wavelength‐dependent,^[^ ^13^ ^]^ the use of molecularly tailored UCNPs that are excited upon NIR illumination and emit light in the visible spectrum enables transcranial deep brain stimulation by red‐shifting excitation wavelengths. During the synthesis process, these core–shell UCNPs were coated with silica or poly(acrylic acid) to improve their long‐term stability and biocompatibility.^[^ ^37^ ^]^ Notably, NIR light generated outside the skull at a distance of several millimeters was shown to trigger dopamine release from genetically tagged neurons. To date, most of the in vivo studies investigating nanotechnology‐enabled optical modulation were performed on rodent models. A key step toward their clinical applications will be implementation in non‐human primates, which will require changes in strategy in the context of the larger size of the primate brain.^[^ ^38^ ^]^ Moreover, further characterization of the metabolic and immune responses to UCNPs will be needed. Despite the benefits of NIR stimulation, UCNPs usually require high power NIR light source^[^ ^39^ ^]^ with power density ranging from 1.1 to 21.7 mW mm^−2^.^[^ ^40^, ^41^ ^]^

While genetic modifications are widely adopted in research studies on rodents, they remain limited in non‐human primates and restricted in clinical settings. Genetic manipulation via viral tools is needed for optogenetics. However, the associated safety concerns make the applications and translation of optogenetics from rodents to non‐human primates and clinical studies challenging. In this regard, emerging forms of nanostructures provide novel strategies for non‐genetic optical neuromodulation through targeted and localized photothermal effect without the need for genetic modifications. For example, Au nanoparticles can be conjugated to high‐avidity ligands of membrane proteins of neurons for extracellular neuromodulation.^[^ ^42^ ^]^ In this scheme, targeting cells of interest is achieved by conjugating Au nanoparticles to synthetic molecules or antibodies that specifically bind to membrane proteins. This strategy requires cell‐type‐identifying membrane proteins and is more limited than genetic methods in this regard. Further investigation is helpful to assess the efficacy and selectivity of this strategy in vivo. Moreover, nanostructures such as extracellular Si mesostructures^[^ ^43^ ^]^ and intracellular Si nanowires^[^ ^44^ ^]^ can produce pronounced photothermal effect upon light irradiation that induces localized temperature increase, transiently opens ion channels and triggers actional potentials. The internalized Si nanowires provide a platform for light‐controlled non‐genetic neuromodulation of neural activity through a glial‐mediated pathway.^[^ ^44^ ^]^ In addition, poly(3,4‐ethylenedioxythiophene) polystyrene sulfonate (PEDOT:PSS)‐based electrochromic thin film with thickness on the nanometer scale was recently reported to photothermally stimulate and electrochemically record cultured neurons and brain slices,^[^ ^45^ ^]^ which represents a new paradigm of optical interrogation without genetic or molecular manipulation.

### 3.3 Acoustic Neuromodulation

Acoustic neuromodulation using FUS offers a unique combination of advantages in noninvasiveness, deep brain penetration and sharp spatial focus. FUS is able to focally concentrate energy and effectively deliver power and signals to devices.^[^ ^46^ ^]^ However, due to the bulky size of FUS neural stimulators, in vivo animal experiments have long been limited to acute sessions under anesthesia and body fixation. Recently, the use of nanofabrication leads to miniaturized head‐mounted FUS stimulators and thereby enables chronic experiments on freely behaving animals (Figure 2c).^[^ ^47^ ^]^ The lightweight neural stimulator could be synchronized with electromyography recording system to enable closed‐loop neuromodulation. FUS can penetrate into tissue deeper than 1 cm.^[^ ^46^ ^]^ A notable report seeking to understand the mechanism for FUS‐induced cortical response used the following modulation parameters: frequency of 500 kHz, pulse duration of 200 µs and intensities between 0.034 and 4.2 W cm^−2^.^[^ ^5^ ^]^ The frequency is above the range of hearing of mice, but secondary auditory effects were observed. FUS neuromodulation is also a relatively gentle technique without tissue heating or other visible damage to the target brain region.^[^ ^48^ ^]^ Tissues that underwent low‐intensity FUS modulation appeared as healthy as control tissues without stimulation.

While acoustic signals prove accurate in measuring and modulating brain activity in the short term, it is challenging to deploy them on a long‐term basis because they require the application of an impedance matching gel as the liquid medium on the skin, which will dry out within hours. Another challenge is related to the direction of external and internal devices where any migration/motion will affect the performance of acoustic modulation. Furthermore, acoustic signals for neuromodulation may induce cavitational damage in the tissue.^[^ ^49^ ^]^ Continuous exposure to acoustic signals was shown to damage brain development in mouse embryos.^[^ ^50^ ^]^ Thus, potential hazard due to the continuous, long‐term exposure to FUS at different stages of the brain development needs to be evaluated thoroughly. With ongoing research into the miniaturization of head‐mounted neuromodulation systems, power needs will dictate the size of the overall system.

### 3.4 Chemical Neuromodulation

Conventional chemogenetic approaches, such as the widely used DREADD technology,^[^ ^4^ ^]^ enable spatial and temporal control of specific neural signaling pathways. However, as noted above, one major drawback that limits the use of chemogenetics on behavioral studies thus far has been the low temporal resolution in the minutes–hours timeframe needed for systemic administration and GPCR activation. Recognizing the potential of nanoclusters in catalyzing neuronal signaling pathways and achieving more localized neuromodulation with controllable kinetics, iron sulfide‐based nanocatalysts that catalyze the local generation of nitric oxide (NO) were recently developed.^[^ ^51^ ^]^ This gaseous molecule activates the transient receptor potential cation channel subfamily V member 1 (TRPV1) (Figure 2d). Notably, the latency of TRPV1‐mediated neuromodulation is on the order of 100 s, and is quantitatively controllable by changing the applied voltage. This electrocatalytic NO generation platform implemented in an implantable probe allows for neuromodulation in the targeted regions in the CNS.

### 3.5 Magnetic Neuromodulation

Magnetic neuromodulation represents a powerful noninvasive neurostimulation method, although it still suffers from the attenuation through neural tissue and low focusing capacity. Recent years have witnessed multiple lines of effort in addressing these limitations by nanotechnology. For example, μ‐magnetic stimulation (μMS) has been introduced as an implantable alternative to TMS, with improved resolution.^[^ ^52^ ^]^ However, selective stimulation still presents a challenge. A new silicon‐based µ‐coil design has been presented its effectiveness in activating cortical pyramidal neurons in vitro as well as driving behavioral responses in vivo.^[^ ^53^, ^54^ ^]^ An asymmetric magnetic field has increased directionality, preventing simultaneous activation of nearby axons and can provide a spatial resolution of 60 μm. Furthermore, as magnetic fields are permeable through encapsulation and glial scar, the performance of implantable µ‐coils does not degrade as stiff electrodes would, since electrical stimulation requires close contact with target neurons. A design of a copper coil encompassing quartz at the core^[^ ^55^ ^]^ was elaborated by finite element modelling and fabricated for in vitro testing. Action potentials were elicited from retinal ganglion cells at a distance of up to 1100 μm, with marked increases in the number of spikes recorded when the coil was placed along the plane of the cells or the stimulation amplitude was increased. Modulation of the field strength may be accomplished by altering these two parameters. Highly conductive metals such as copper and silver are the most common materials for fabricating magnetic microcoils; however, they are considered toxic for biological use.^[^ ^56^ ^]^ Encapsulating these materials with a layer of parylene‐C, SiN_*x*_ or SiO_2_ is a key step toward fully biocompatible microcoils.

On the other hand, magnetic nanoparticles represent a significant portion of magnetic neuromodulation development. Superparamagnetism—a form of magnetism appearing in magnetic nanoparticles—is a representative example of the unique properties that occur at the nanoscale. Superparamagnetic nanoparticles can be delivered to deep brain structures and remotely controlled, enabling an effective strategy to achieve deep brain stimulation. Force‐generating or heat‐dissipating superparamagnetic nanoparticles can be used for wireless neuromodulation by modulating specific ion channels.^[^ ^57^, ^58^, ^59^, ^60^ ^]^ The ion channels of specific cells are targeted, either by exploiting their natural action, or through genetic modification. Mechanosensitive channels (e.g., TREK1^[^ ^57^ ^]^ and Piezo1^[^ ^58^ ^]^) are activated by the magnetic force of synthetic magnetic nanoparticles, while heat‐sensitive ion channels (e.g., TRPV1) can be activated through magnetic nanoparticles that generate heat in response to an external alternating magnetic field (Figure 2e).^[^ ^59^, ^60^ ^]^ Importantly, there is no observable difference in neuronal density and glial response between stimulated and unstimulated subjects, indicating that the transiently dissipated heat generated by the magnetic nanoparticles result in negligible tissue damage.^[^ ^60^ ^]^

Magnetogenetics, the magnetic counterpart of optogenetics, chemogenetics, and sonogenetics, uses magnetic stimuli to manipulate cell behavior via magnetoreceptors. Single magnetic proteins such as MagR have been used to stimulate the neurons, but further investigation is required to understand the operating principle, the mechanism of coupling with ion channels, etc.^[^ ^61^ ^]^ While magnetogenetics can be less invasive compared to optogenetics, the magnetic forces generated by previously reported iron‐containing proteins are inadequate to produce significant magnetic effects. The energy produced from these magnetic proteins is much lower than the thermal energy generated from the magnetic nanoparticles.^[^ ^62^ ^]^ Recognizing this limitation, efforts have been made to engineer genetically encoded magnetic protein crystal.^[^ ^63^ ^]^ Notably, the engineered protein crystal can generate magnetic forces 9 orders of magnitude larger than those used in previous studies, paving the way for exploiting the full potential of magnetogenetics. A different mode of magnetogenetic modulation is via ion channels gated by magnetic heating. However, quantitative analyses suggest that it has certain physical limits.^[^ ^62^ ^]^ The surface temperature of 6 nm MnFe_2_O_4_ nanoparticles has been shown to increase by more than 4 °C under a radio‐frequency magnetic field, raising the temperature of nearby cell membranes to ≈40 °C, at which point spikes with action potential magnitude were observed.^[^ ^59^ ^]^ 22 nm Fe_3_O_4_ nanoparticles increase in temperature to above 43 °C under an alternating current (AC) magnetic field, above the activation temperature for TRPV1.^[^ ^60^ ^]^ By contrast, ferritin protein with a 6 nm iron core, which is of comparable size to MnFe_2_O_4_, has negligible heating capabilities. In general, the heating efficiency of magnetic nanoparticles strongly depends on their size, and those with a diameter below 10 nm have very low heating efficiency. Its theoretical maximum temperature increase is merely 1.5 × 10^–10^ K even when modified to boost its specific heating rate.^[^ ^62^ ^]^

## 4 Nanomaterial‐Mediated Cross‐Modal Neuromodulation

Magnetic and acoustic neuromodulation modalities excel in penetration depth and noninvasiveness, while electrical, optical, and chemical neuromodulation modalities have strengths in localized stimulation with high precision. In addition to addressing limitations of each individual modality as discussed above, nanomaterials and nanoscale devices also enable emerging forms of graft neuromodulation modalities by being employed as signal transducers across energy terms. Acting as mediators, these nanoscale platforms are capable of harnessing and converting a primary stimulus, typically a wirelessly and remotely transmitted, noninvasive, deep‐penetrating signal such as ultrasonic or magnetic stimulus, to a secondary stimulus, typically a physiologically relevant signal such as electric, optical or chemical stimulus at the localized neural interface. By grafting multiple neuromodulation modalities, these transduction schemes can integrate the strengths and circumvent respective limitations of individual modalities to provide improved neuromodulation capabilities. While we acknowledge the elegant demonstrations using nanomaterials and nanoscale devices for multimodal neuromodulation “A + B” (such as electrical + chemical^[^ ^24^ ^]^ and optical + chemical^[^ ^64^, ^65^, ^66^ ^]^), we focus our discussion on cross‐modal neuromodulation “A to B”.

### 4.1 Ultrasound as Input Modality for Cross‐Modal Neuromodulation

#### 4.1.1 Acousto‐Electric Neuromodulation

**Figure 3:**
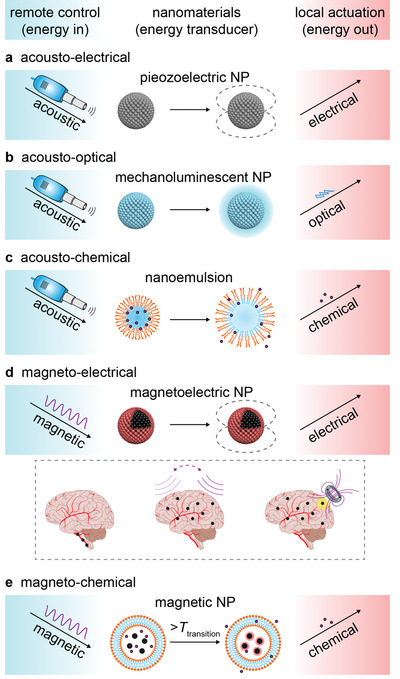
Nanomaterial‐mediated cross‐modal modulation. a) Acousto‐electrical neuromodulation using piezoelectric nanoparticles. b) Acousto‐optical neuromodulation using mechanoluminescent nanoparticles. c) Acousto‐chemical neuromodulation through nanoemulsion‐assisted release of chemical compound. d) Magneto‐electrical neuromodulation using magnetoelectric nanoparticles. e) Magneto‐chemical neuromodulation through magnetic nanoparticle‐triggered release of chemical compound.

Piezoelectric materials are solid materials that can convert acoustic waves into electric fields via acoustoelectric transduction. Nanoscale piezoelectric materials can be used to locally modulate neural activity by activating voltage‐gated ion channels with electric fields. Notably, nanotechnology leads to a substantial volume reduction of lead zirconate titanate (PZT)‐based piezoelectric transducers and thereby enables wireless, untethered and battery‐free implantable acoustic neural stimulators of rat sciatic nerve.^[^ ^67^ ^]^ The miniaturized neural stimulator incorporating piezoelectric transducer, an energy‐storage capacitor and an integrated circuit has the potential to facilitate powerful therapeutic interventions through closed‐loop neuromodulation. On the other hand, other lead‐free piezoelectric materials have also been explored for neuromodulation studies given the safety considerations of PZT. For example, piezoelectric barium titanate nanoparticles have been used as transducers to stimulate neuron‐like cells^[^ ^68^ ^]^ (**Figure** **3**a), showing their potential for noninvasive acoustic neuromodulation. Future investigation using animal models is needed to assess their feasibility in vivo, and effort in further improving piezoelectric coefficients of these substitute materials is needed to provide better piezoelectric performance.

#### 4.1.2 Acousto‐Optical Neuromodulation

The major drawbacks of in vivo optogenetic modulation include the limited penetration depth of visible light and the intrusive surgical implantation of tethered optical fibers. While the use of NIR light for optogenetics increases the penetration depth by a few millimeters,^[^ ^37^ ^]^ it is still inherently limited by the scattering in lipid‐rich brain tissue and not on par with ultrasound. Therefore, it is beneficial to leverage the wirelessly transmitted, noninvasive, deep penetrating ultrasound with optogenetics to enable a new paradigm acousto‐optical neuromodulation.^[^ ^69^ ^]^ In this graft neuromodulation modality, FUS has strengths of penetration depth and spatial focusing, while optogenetics provides the cell‐type specificity. Mechanoluminescent ZnS:Ag,Co@ZnS nanoparticles function as local light sources for optogenetic neuromodulation when triggered by FUS through the intact skull of adult mouse (Figure 3b). Notably, the nanoscale light emitters can be recharged repetitively in superficial blood vessels by excitation light as they circulate within the systemic circulation.

#### 4.1.3 Acousto‐Chemical Neuromodulation

Nanoparticle carriers can be employed to noninvasively uncage neuromodulatory drugs under sonication, thereby enabling a remotely controlled chemical modulation. Upon FUS application, a liquid perfluorocarbon core encapsulated by a block copolymer matrix shell undergoes a liquid‐to‐gas phase transition and releases the drug cargo^[^ ^70^ ^]^ (Figure 3c). In this proof‐of‐principle in vivo study using an acute rat seizure model, the nanoparticles show strong efficacy in silencing seizures by uncaging a small molecule lipophilic anesthetic that can cross the blood–brain barrier (BBB) without the need for disruption. This FUS‐gated nanoparticle‐mediated drug release platform shows potential for noninvasive targeted neuromodulation in basic and clinical neuroscience. This remotely controlled drug release platform could be next used to test clinically approved drugs.^[^ ^71^ ^]^

### 4.2 Magnetic Field as Input Modality for Cross‐Modal Neuromodulation

#### 4.2.1 Magneto‐Electric Neuromodulation

Similar to acoustic waves, the magnetic field is another form of energy with the advantage of wireless transmission and deep penetration, and therefore has been widely employed as a primary stimulus for noninvasive remote control. A wide range of magnetic nanoparticles have been used as signal transducers for magnetoelectric neuromodulation. Magnetoelectric nanoparticles (MENs) are commonly derived from a CoFe_2_O_4_–BaTiO_3_ heterostructure and can be injected into live animals for neuronal stimulation. CoFe_2_O_4_–BaTiO_3_ core–shell nanoparticles were used in a proof‐of‐principle study to stimulate specific neural circuits deep in the mouse brain under a low‐intensity magnetic field.^[^ ^72^ ^]^ Moreover, selective control and wireless navigation deep in the CNS of GCaMP6s transgenic mice while maintaining external control via the magnetic field were demonstrated.^[^ ^73^ ^]^ Neural responses were consistently recorded with a short latency period through calcium imaging of acute and organotypic slices. As shown in Figure 3d, the MENs could be delivered into the bloodstream and guided to cross into the CNS by an external direct current (DC) magnet. Wireless stimulation may be carried out upon application of an external alternating magnetic field without inducing a noticeable neuroinflammatory response in the brain. Most recently, CoFe_2_O_4_–BaTiO_3_ nanoparticles have also been successfully demonstrated to modulate subthalamic region deep in the brain of freely behaving mice.^[^ ^74^ ^]^

Small volumes (1 mg of particles in 0.2 mL of liquid) of MENs are sufficient to stimulate single neurons with an AC magnetic field of 3.58 × 10^4^ A m^−1^ at 10 Hz.^[^ ^73^ ^]^ While this is a promising in vivo proof of concept, the level of magnetic field allowable for head and chest exposure as defined by the IEEE is (1.44 × 10^4^)/*f* A m^−1[^ ^75^ ^]^ which equates to 1.44 × 10^3^ A m^−1^ at 10 Hz. Larger magneto‐electric neuromodulation systems that utilize implantable magneto‐electric wireless power transfer can achieve sufficient power for stimulation at less than 1 mT and frequencies higher than 100 kHz.^[^ ^76^ ^]^ Improving magneto‐electric coefficient of nanoparticles and investigating the lowest possible magneto‐electric field for neuromodulation will make this technology more feasible for human implementation.

In addition to the substantial volume reduction, nanoparticles can be readily combined with biocompatible polymers or hydrogels to further reduce the mechanical mismatch between the neuromodulator and target tissue.^[^ ^77^ ^]^ Coatings such as glycerol mono‐oleate have been employed to reduce cytotoxicity and ensure the uniform dispersion of nanoparticles.^[^ ^73^ ^]^ A hydrogel structure diffused with CoFe_2_O_4_–BiFeO_3_ core–shell particles may also be directed toward target neurons using a rotating magnetic field. Tested with SH‐SY5Y cells, the hydrogel‐MEN devices dispatch their load of living cells as it biodegrades, and stimulate nearby neurons as a result of the magneto‐electric effect. Magnetoelectric stimulation was also employed to facilitate neuronal differentiation for developing models of Parkinson's disease. Looking forward, further research assessing the long‐term in vivo efficacy of this hydrogel‐MEN platform would be necessary.

The required magnetic field strength, which is strictly regulated for human tissue exposure, is much lower for nanoscale neuromodulation techniques compared to repetitive TMS (rTMS). While both therapies operate largely in the 0–20 Hz range, rTMS requires a field strength on the order of 10^4^ Oe,^[^ ^72^ ^]^ while nanoparticle approaches may require only 100s of Oe.^[^ ^72^, ^73^ ^]^ The exposure limit, as set by the International Commission on Non‐Ionizing Radiation Protection (ICNIRP), is dependent on frequency.^[^ ^78^ ^]^ In the 1–8 Hz frequency range, the limit is around 400 Oe for continuous public exposure. Large‐magnitude AC magnetic fields may cause tissue heating and other adverse side effects. The low‐strength magnetic fields employed by nanoparticle therapies are a significant benefit when compared to high‐strength rTMS.

#### 4.2.2 Magneto‐Chemical Neuromodulation

One major drawback limiting the use of conventional chemogenetic approaches has been the low temporal resolution in the minutes–hours timeframe needed for systemic administration. To improve temporal and spatial precision, magnetic field‐responsive nanomaterials can be used for local delivery of neuromodulators. In response to alternating magnetic fields, magnetic nanoparticles injected into freely moving mice dissipate heat, and subsequently trigger the release of chemical neuromodulators from thermally sensitive liposomes^[^ ^79^ ^]^ (Figure 3e). This magneto‐chemical neuromodulation strategy achieves a remotely controlled, wireless, noninvasive and precise chemical manipulation of targeted neural circuits with a latency reduced to 20 s, with the potential to be further improved by optimizing the heating efficiency of magnetic nanoparticles.

## 5 Outlook

Recent advances in nanotechnology are revolutionizing neuroscience studies by enabling novel modalities of neuromodulation.^[^ ^9^ ^]^ The nanotechnology toolkit continues to blur the distinctions between artificial and biological systems through the miniaturization of material structures and device architectures to micro/nanoscale. Nanomaterials and nanoscale devices offer the opportunities to interrogate specific cell populations in the brain in a less invasive manner with unprecedented spatiotemporal resolution. Their capability to seamlessly integrate with the nervous system and efficiently transduce signals across energy terms could shape the future of neuromodulation therapy. Moving forward, continued work can open up new possibilities for next‐generation neuromodulation modalities for fundamental neuroscience research and clinical translation.

### 5.1 Neuromodulation for Neuroscience Research

First and foremost, we envision that cross‐modal modulation strategies will continue gaining interest as these combinational modalities provide optimized routes for neuromodulation by taking advantages of the strengths while bypassing the limitations of individual modalities. The diverse selection of combinational modalities depends largely on the specific neuromodulation application as well as the accessibility of target cell populations.

Second, while the dramatic reduction in dimension and footprint of nanoscale platforms leads to significantly less invasive neuromodulation strategies, bioengineering approaches are needed to make these strategies entirely noninvasive. Recently, there have been multiple efforts to develop noninvasive gene delivery methods for neuromodulation, including the use of viral variants capable of crossing the BBB,^[^ ^80^ ^]^ transient BBB opening via FUS,^[^ ^81^ ^]^ and using genetically modified mouse lines for optogenetics^[^ ^69^ ^]^ and chemogenetics.^[^ ^82^ ^]^

Third, precise regulation of neural functions in an autonomous, programmable, closed‐loop manner would take neuromodulation a step closer to personalized medicine. By integrating real‐time sensing modalities, devices can adjust the intensity of neuromodulation in dynamic response to physiological and pathological neural activity in the local environment. These sophisticated bidirectional communication capabilities to transmit information to and from the brain lay the foundation for precise and personalized treatments for a variety of conditions such as Parkinson's disease and epilepsy.

Fourth, next‐generation neuromodulation would benefit greatly from synergy among neurotechnology, tissue engineering, data science, and artificial intelligence^[^ ^83^ ^]^—research disciplines that have long been disassociated from each other. For example, the symbiotic coexistence of artificial neuromorphic devices integrated with engineered brain tissue may drive unprecedented graft–host interactions toward integration and dynamic adaptation to rebuild malfunctioning brain circuits. Moreover, high‐frequency data collection and processing of stimulation/recording signals from high‐density arrays of thousands of miniaturized active channels in parallel over long‐term behavioral sessions becomes increasingly challenging. A real‐time closed‐loop feedback control system with detection and modulation capabilities necessitates optimization for data storage and decoding hardware, as well as efficient software algorithms such as machine learning that incorporate biological insights.

### 5.2 Neuromodulation for Clinical Application

While emerging neuromodulation modalities have demonstrated to be potent research tools in animal studies, certain challenges remain to be overcome on its path to clinical implementation for diagnostics, prosthetics, and therapeutics.

First, comprehensive investigations on the safety and chronic biocompatibility of these nanoscale platforms would constitute a critical step in evaluating their future clinical translation. For the wide variety of functional nanoparticles, detailed characterizations and rigorous statistical analyses about their diffusion and metabolism dynamics will shed light on the cellular and tissue response and potential toxicity. Thermal effects are another consideration for assessing ultrasonic and magnetic neuromodulation modalities that trigger heat‐sensitive ion channels. In addition, addressing the uncertainties in the safety and efficacy for use of genetic manipulations in humans would be essential for the translational development of optogenetics, chemogenetics, sonogenetics, magnetogenetics, and their derivative strategies.

Second, realizing the full potential of these appealing neuromodulation modalities in clinical settings calls for a convergence of efforts from not only the research community, but also industry. Collaboration between academy and industry (e.g., Neuralink^[^ ^84^ ^]^) will accelerate the development and promote the dissemination of these emerging technologies as widely accessible tools in healthcare facilities.

In summary, the vibrant field of nanotechnology has brought and will continue bringing new vigor to its intersection with neuroscience for next‐generation neuromodulation modalities.

## Conflict of Interest

The authors declare no conflict of interest.

## References

[1] International Neuromodulation Society, https://www.neuromodulation.com/ (accessed: April 2021).

[2] Editorial , Nat. Biotechnol. 2019, 37, 967. 31485053 10.1038/s41587-019-0263-3

[3] L. Z. Fan , S. Kheifets , U. L. Böhm , H. Wu , K. D. Piatkevich , M. E. Xie , V. Parot , Y. Ha , K. E. Evans , E. S. Boyden , A. E. Takesian , A. E. Cohen , Cell 2020, 180, 521. 31978320 10.1016/j.cell.2020.01.001 PMC7259440

[4] B. L. Roth , Neuron 2016, 89, 683. 26889809 10.1016/j.neuron.2016.01.040 PMC4759656

[5] T. Sato , M. G. Shapiro , D. Y. Tsao , Neuron 2018, 98, 1031. 29804920 10.1016/j.neuron.2018.05.009 PMC8127805

[6] W. Legon , T. F. Sato , A. Opitz , J. Mueller , A. Barbour , A. Williams , W. J. Tyler , Nat. Neurosci. 2014, 17, 322. 24413698 10.1038/nn.3620

[7] S. C. Murphy , L. M. Palmer , T. Nyffeler , R. M. Müri , M. E. Larkum , eLife 2016, 5, e13598. 26988796 10.7554/eLife.13598 PMC4811769

[8] F. B. Wagner , J.‐B. Mignardot , C. G. Le Goff‐Mignardot , R. Demesmaeker , S. Komi , M. Capogrosso , A. Rowald , I. Seáñez , M. Caban , E. Pirondini , M. Vat , L. A. McCracken , R. Heimgartner , I. Fodor , A. Watrin , P. Seguin , E. Paoles , K. Van Den Keybus , G. Eberle , B. Schurch , E. Pralong , F. Becce , J. Prior , N. Buse , R. Buschman , E. Neufeld , N. Kuster , S. Carda , J. von Zitzewitz , V. Delattre , T. Denison , H. Lambert , K. Minassian , J. Bloch , G. Courtine , Nature 2018, 563, 65. 30382197 10.1038/s41586-018-0649-2

[9] Y. Temel , A. Jahanshahi , Science 2015, 347, 1418. 25814569 10.1126/science.aaa9610

[10] A. M. Lozano , N. Lipsman , H. Bergman , P. Brown , S. Chabardes , J. W. Chang , K. Matthews , C. C. McIntyre , T. E. Schlaepfer , M. Schulder , Y. Temel , J. Volkmann , J. K. Krauss , Nat. Rev. Neurol. 2019, 15, 148. 30683913 10.1038/s41582-018-0128-2 PMC6397644

[11] E. S. Boyden , F. Zhang , E. Bamberg , G. Nagel , K. Deisseroth , Nat. Neurosci. 2005, 8, 1263. 16116447 10.1038/nn1525

[12] K. Deisseroth , Nat. Methods 2011, 8, 26. 21191368 10.1038/nmeth.f.324 PMC6814250

[13] G. Hong , A. L. Antaris , H. Dai , Nat. Biomed. Eng. 2017, 1, 0010.

[14] R. Chen , F. Gore , Q.‐A. Nguyen , C. Ramakrishnan , S. Patel , S. H. Kim , M. Raffiee , Y. S. Kim , B. Hsueh , E. Krook‐Magnusson , I. Soltesz , K. Deisseroth , Nat. Biotechnol. 2021, 39, 161. 33020604 10.1038/s41587-020-0679-9 PMC7878426

[15] J. Kubanek , P. Shukla , A. Das , S. A. Baccus , M. B. Goodman , J. Neurosci. 2018, 38, 3081. 29463641 10.1523/JNEUROSCI.1458-17.2018 PMC5864152

[16] M. Hallett , Nature 2000, 406, 147. 10910346 10.1038/35018000

[17] Y.‐S. Huang , C.‐H. Fan , N. Hsu , N.‐H. Chiu , C.‐Y. Wu , C.‐Y. Chang , B.‐H. Wu , S.‐R. Hong , Y.‐C. Chang , A. Yan‐Tang Wu , V. Guo , Y.‐C. Chiang , W.‐C. Hsu , L. Chen , C. Pin‐Kuang Lai , C.‐K. Yeh , Y.‐C. Lin , Nano Lett. 2020, 20, 1089. 31884787 10.1021/acs.nanolett.9b04373

[18] Transcranial Magnetic Stimulation: Clinical Applications for Psychiatric Practice, (Eds: R. A. Bermudes , K. I. Lanocha , P. G. Janicak ), American Psychiatric Association Publishing, Arligton, VA, USA 2017.

[19] Repetitive Transcranial Magnetic Stimulation (rTMS) Systems – Class II Special Controls Guidance for Industry and FDA Staff, https://www.fda.gov/medical-devices/guidance-documents-medical-devices-and-radiation-emitting-products/repetitive-transcranial-magnetic-stimulation-rtms-systems-class-ii-special-controls-guidance (accessed: June 2021).

[20] M. Lu , S. Ueno , PLoS One 2017, 12, e0178422. 28586349 10.1371/journal.pone.0178422 PMC5460812

[21] R. Das , F. Moradi , H. Heidari , IEEE Trans. Biomed. Circuits Syst. 2020, 14, 343. 31944987 10.1109/TBCAS.2020.2966920

[22] H. Acarón Ledesma , X. Li , J. L. Carvalho‐de‐Souza , W. Wei , F. Bezanilla , B. Tian , Nat. Nanotechnol. 2019, 14, 645. 31270446 10.1038/s41565-019-0487-x PMC6800006

[23] X. Yang , T. Zhou , T. J. Zwang , G. Hong , Y. Zhao , R. D. Viveros , T.‐M. Fu , T. Gao , C. M. Lieber , Nat. Mater. 2019, 18, 510. 30804509 10.1038/s41563-019-0292-9 PMC6474791

[24] I. R. Minev , P. Musienko , A. Hirsch , Q. Barraud , N. Wenger , E. M. Moraud , J. Gandar , M. Capogrosso , T. Milekovic , L. Asboth , R. F. Torres , N. Vachicouras , Q. Liu , N. Pavlova , S. Duis , A. Larmagnac , J. Voeroes , S. Micera , Z. Suo , G. Courtine , S. P. Lacour , Science 2015, 347, 159. 25574019 10.1126/science.1260318

[25] E. McGlynn , V. Nabaei , E. Ren , G. Galeote‐Checa , R. Das , G. Curia , H. Heidari , Adv. Sci. 2021, 8, 2002693. 10.1002/advs.202002693 PMC8132070 34026431

[26] J. W. Salatino , K. A. Ludwig , T. D. Y. Kozai , E. K. Purcell , Nat. Biomed. Eng. 2017, 1, 862. 30505625 10.1038/s41551-017-0154-1 PMC6261524

[27] R. Chen , A. Canales , P. Anikeeva , Nat. Rev. Mater. 2017, 2, 16093. 31448131 10.1038/natrevmats.2016.93 PMC6707077

[28] R. Feiner , T. Dvir , Nat. Rev. Mater. 2017, 3, 17076.

[29] T.‐M. Fu , G. Hong , T. Zhou , T. G. Schuhmann , R. D. Viveros , C. M. Lieber , Nat. Methods 2016, 13, 875. 27571550 10.1038/nmeth.3969

[30] N. Vachicouras , O. Tarabichi , V. V. Kanumuri , C. M. Tringides , J. Macron , F. Fallegger , Y. Thenaisie , L. Epprecht , S. McInturff , A. A. Qureshi , V. Paggi , M. W. Kuklinski , M. C. Brown , D. J. Lee , S. P. Lacour , Sci. Transl. Med. 2019, 11, eaax9487. 31619546 10.1126/scitranslmed.aax9487

[31] F. Patolsky , B. P. Timko , G. Yu , Y. Fang , A. B. Greytak , G. Zheng , C. M. Lieber , Science 2006, 313, 1100. 16931757 10.1126/science.1128640

[32] Y. Zhao , S. S. You , A. Zhang , J.‐H. Lee , J. Huang , C. M. Lieber , Nat. Nanotechnol. 2019, 14, 783. 31263191 10.1038/s41565-019-0478-y

[33] J. Abbott , T. Ye , K. Krenek , R. S. Gertner , S. Ban , Y. Kim , L. Qin , W. Wu , H. Park , D. Ham , Nat. Biomed. Eng. 2019, 4, 232. 31548592 10.1038/s41551-019-0455-7 PMC7035150

[34] S. P. Pașca , Nature 2018, 553, 437. 29364288 10.1038/nature25032

[35] S. P. Paşca , Science 2019, 363, 126. 30630918 10.1126/science.aau5729

[36] T.‐i. Kim , J. G. McCall , Y. H. Jung , X. Huang , E. R. Siuda , Y. Li , J. Song , Y. M. Song , H. A. Pao , R.‐H. Kim , C. Lu , S. D. Lee , I.‐S. Song , G. Shin , R. Al‐Hasani , S. Kim , M. P. Tan , Y. Huang , F. G. Omenetto , J. A. Rogers , M. R. Bruchas , Science 2013, 340, 211. 23580530 10.1126/science.1232437 PMC3769938

[37] S. Chen , A. Z. Weitemier , X. Zeng , L. He , X. Wang , Y. Tao , A. J. Y. Huang , Y. Hashimotodani , M. Kano , H. Iwasaki , L. K. Parajuli , S. Okabe , D. B. L. Teh , A. H. All , I. Tsutsui‐Kimura , K. F. Tanaka , X. Liu , T. J. McHugh , Science 2018, 359, 679. 29439241 10.1126/science.aaq1144

[38] Y. Shen , R. E. Campbell , D. C. Côté , M.‐E. Paquet , Front. Neural Circuits 2020, 14, 41. 32760252 10.3389/fncir.2020.00041 PMC7373823

[39] N. Yu , L. Huang , Y. Zhou , T. Xue , Z. Chen , G. Han , Adv. Healthcare Mater. 2019, 8, 1801132. 10.1002/adhm.201801132 30633858

[40] Z. Wang , M. Hu , X. Ai , Z. Zhang , B. Xing , Adv. Biosyst. 2019, 3, 1800233. 10.1002/adbi.201800233 32627341

[41] X. Wu , Y. Zhang , K. Takle , O. Bilsel , Z. Li , H. Lee , Z. Zhang , D. Li , W. Fan , C. Duan , E. M. Chan , C. Lois , Y. Xiang , G. Han , ACS Nano 2016, 10, 1060. 26736013 10.1021/acsnano.5b06383 PMC4913696

[42] J. L. Carvalho‐de‐Souza , J. S. Treger , B. Dang , S. B. H. Kent , D. R. Pepperberg , F. Bezanilla , Neuron 2015, 86, 207. 25772189 10.1016/j.neuron.2015.02.033 PMC4393361

[43] Y. Jiang , J. L. Carvalho‐de‐Souza , R. C. S. Wong , Z. Luo , D. Isheim , X. Zuo , A. W. Nicholls , I. W. Jung , J. Yue , D.‐J. Liu , Y. Wang , V. De Andrade , X. Xiao , L. Navrazhnykh , D. E. Weiss , X. Wu , D. N. Seidman , F. Bezanilla , B. Tian , Nat. Mater. 2016, 15, 1023. 27348576 10.1038/nmat4673 PMC5388139

[44] Y. Jiang , X. Li , B. Liu , J. Yi , Y. Fang , F. Shi , X. Gao , E. Sudzilovsky , R. Parameswaran , K. Koehler , V. Nair , J. Yue , K. Guo , Y. Fang , H.‐M. Tsai , G. Freyermuth , R. C. S. Wong , C.‐M. Kao , C.‐T. Chen , A. W. Nicholls , X. Wu , G. M. G. Shepherd , B. Tian , Nat. Biomed. Eng. 2018, 2, 508. 30906646 10.1038/s41551-018-0230-1 PMC6430241

[45] F. S. Alfonso , Y. Zhou , E. Liu , A. F. McGuire , Y. Yang , H. Kantarci , D. Li , E. Copenhaver , J. B. Zuchero , H. Müller , B. Cui , Proc. Natl. Acad. Sci. U. S. A. 2020, 117, 17260. 32632007 10.1073/pnas.2002352117 PMC7382226

[46] C. Rabut , S. Yoo , R. C. Hurt , Z. Jin , H. Li , H. Guo , B. Ling , M. G. Shapiro , Neuron 2020, 108, 93. 33058769 10.1016/j.neuron.2020.09.003 PMC7577369

[47] H. Kim , S. Kim , N. S. Sim , C. Pasquinelli , A. Thielscher , J. H. Lee , H. J. Lee , Brain Stimul. 2019, 12, 251. 30503712 10.1016/j.brs.2018.11.007

[48] R. F. Dallapiazza , K. F. Timbie , S. Holmberg , J. Gatesman , M. B. Lopes , R. J. Price , G. W. Miller , W. J. Elias , J. Neurosurg. 2018, 128, 875. 28430035 10.3171/2016.11.JNS16976 PMC7032074

[49] Y. Tufail , A. Yoshihiro , S. Pati , M. M. Li , W. J. Tyler , Nat. Protoc. 2011, 6, 1453. 21886108 10.1038/nprot.2011.371

[50] E. S. B. C. Ang , V. Gluncic , A. Duque , M. E. Schafer , P. Rakic , Proc. Natl. Acad. Sci. USA 2006, 103, 12903. 16901978 10.1073/pnas.0605294103 PMC1538990

[51] J. Park , K. Jin , A. Sahasrabudhe , P.‐H. Chiang , J. H. Maalouf , F. Koehler , D. Rosenfeld , S. Rao , T. Tanaka , T. Khudiyev , Z. J. Schiffer , Y. Fink , O. Yizhar , K. Manthiram , P. Anikeeva , Nat. Nanotechnol. 2020, 15, 690. 32601446 10.1038/s41565-020-0701-x PMC7415650

[52] H.‐J. Park , G. Bonmassar , J. A. Kaltenbach , A. G. Machado , N. F. Manzoor , J. T. Gale , Nat. Commun. 2013, 4, 2463. 24030203 10.1038/ncomms3463 PMC3845906

[53] S. W. Lee , F. Fallegger , B. D. F. Casse , S. I. Fried , Sci. Adv. 2016, 2, e1600889. 27957537 10.1126/sciadv.1600889 PMC5148213

[54] S. W. Lee , K. Thyagarajan , S. I. Fried , IEEE. Trans. Biomed. Eng. 2018, 66, 1680. 30369434 10.1109/TBME.2018.2877713 PMC6561646

[55] G. Bonmassar , S. W. Lee , D. K. Freeman , M. Polasek , S. I. Fried , J. T. Gale , Nat. Commun. 2012, 3, 921. 22735449 10.1038/ncomms1914 PMC3621430

[56] K. Thyagarajan , R. A. Lujan , Q. Wang , J. Lu , S. Kor , B. Kakimoto , N. Chang , J. A. Bert , APL Mater. 2021, 9, 011102. 33520428 10.1063/5.0023486 PMC7808331

[57] J. Dobson , Nat. Nanotechnol. 2008, 3, 139. 18654485 10.1038/nnano.2008.39

[58] J.‐u. Lee , W. Shin , Y. Lim , J. Kim , W. R. Kim , H. Kim , J.‐H. Lee , J. Cheon , Nat. Mater. 2021, 20, 1029. 33510447 10.1038/s41563-020-00896-y

[59] H. Huang , S. Delikanli , H. Zeng , D. M. Ferkey , A. Pralle , Nat. Nanotechnol. 2010, 5, 602. 20581833 10.1038/nnano.2010.125

[60] R. Chen , G. Romero , M. G. Christiansen , A. Mohr , P. Anikeeva , Science 2015, 347, 1477. 25765068 10.1126/science.1261821

[61] S. Qin , H. Yin , C. Yang , Y. Dou , Z. Liu , P. Zhang , H. Yu , Y. Huang , J. Feng , J. Hao , Nat. Mater. 2016, 15, 217. 26569474 10.1038/nmat4484

[62] M. Meister , eLife 2016, 5, e17210. 27529126 10.7554/eLife.17210 PMC5016093

[63] T. L. Li , Z. Wang , H. You , Q. Ong , V. J. Varanasi , M. Dong , B. Lu , S. P. Paşca , B. Cui , Nano Lett. 2019, 19, 6955. 31552740 10.1021/acs.nanolett.9b02266 PMC7265822

[64] J.‐W. Jeong , J. G. McCall , G. Shin , Y. Zhang , R. Al‐Hasani , M. Kim , S. Li , J. Y. Sim , K.‐I. Jang , Y. Shi , D. Y. Hong , Y. Liu , G. P. Schmitz , L. Xia , Z. He , P. Gamble , W. Z. Ray , Y. Huang , M. R. Bruchas , J. A. Rogers , Cell 2015, 162, 662. 26189679 10.1016/j.cell.2015.06.058 PMC4525768

[65] A. Canales , X. Jia , U. P. Froriep , R. A. Koppes , C. M. Tringides , J. Selvidge , C. Lu , C. Hou , L. Wei , Y. Fink , P. Anikeeva , Nat. Biotechnol. 2015, 33, 277. 25599177 10.1038/nbt.3093

[66] S. Park , Y. Guo , X. Jia , H. K. Choe , B. Grena , J. Kang , J. Park , C. Lu , A. Canales , R. Chen , Y. S. Yim , G. B. Choi , Y. Fink , P. Anikeeva , Nat. Neurosci. 2017, 20, 612. 28218915 10.1038/nn.4510 PMC5374019

[67] D. K. Piech , B. C. Johnson , K. Shen , M. M. Ghanbari , K. Y. Li , R. M. Neely , J. E. Kay , J. M. Carmena , M. M. Maharbiz , R. Muller , Nat. Biomed. Eng. 2020, 4, 207. 32076132 10.1038/s41551-020-0518-9

[68] A. Marino , S. Arai , Y. Hou , E. Sinibaldi , M. Pellegrino , Y.‐T. Chang , B. Mazzolai , V. Mattoli , M. Suzuki , G. Ciofani , ACS Nano 2015, 9, 7678. 26168074 10.1021/acsnano.5b03162 PMC9003232

[69] X. Wu , X. Zhu , P. Chong , J. Liu , L. N. Andre , K. S. Ong , K. Brinson , A. I. Mahdi , J. Li , L. E. Fenno , H. Wang , G. Hong , Proc. Natl. Acad. Sci. USA 2019, 116, 26332. 31811026 10.1073/pnas.1914387116 PMC6936518

[70] R. D. Airan , R. A. Meyer , N. P. K. Ellens , K. R. Rhodes , K. Farahani , M. G. Pomper , S. D. Kadam , J. J. Green , Nano Lett. 2017, 17, 652. 28094959 10.1021/acs.nanolett.6b03517 PMC5362146

[71] C. J. Magnus , P. H. Lee , J. Bonaventura , R. Zemla , J. L. Gomez , M. H. Ramirez , X. Hu , A. Galvan , J. Basu , M. Michaelides , S. M. Sternson , Science 2019, 364, eaav5282. 30872534 10.1126/science.aav5282 PMC7252514

[72] R. Guduru , P. Liang , J. Hong , A. Rodzinski , A. Hadjikhani , J. Horstmyer , E. Levister , S. Khizroev , Nanomedicine 2015, 10, 2051. 25953069 10.2217/nnm.15.52 PMC4910966

[73] T. Nguyen , J. Gao , P. Wang , A. Nagesetti , P. Andrew , S. Masood , Z. Vriesmann , P. Liang , S. Khizroev , X. Jin , Neurotherapeutics 2021. 10.1007/s13311-021-01071-0. PMC8609092 34131858

[74] K. L. Kozielski , A. Jahanshahi , H. B. Gilbert , Y. Yu , Ö. Erin , D. Francisco , F. Alosaimi , Y. Temel , M. Sitti , Sci. Adv. 2021, 7, eabc4189. 33523872 10.1126/sciadv.abc4189 PMC7806222

[75] IEEE Standard for Safety Levels with Respect to Human Exposure to Electric, Magnetic, and Electromagnetic Fields, 0 Hz to 300 GHz, https://standards.ieee.org/standard/C95_1-2019.html (accessed: June 2021).

[76] Z. Yu , J. C. Chen , F. T. Alrashdan , B. W. Avants , Y. He , A. Singer , J. T. Robinson , K. Yang , IEEE Trans. Biomed. Circuits Syst. 2020, 14, 1241. 33180732 10.1109/TBCAS.2020.3037862 PMC8712272

[77] M. Dong , X. Wang , X. Z. Chen , F. Mushtaq , S. Deng , C. Zhu , H. Torlakcik , A. Terzopoulou , X. H. Qin , X. Xiao , Adv. Funct. Mater. 2020, 30, 1910323.

[78] International Commission on Non‐Ionizing Radiation Protection, *Health Phys*. 2010, 99, 818.

[79] S. Rao , R. Chen , A. A. LaRocca , M. G. Christiansen , A. W. Senko , C. H. Shi , P.‐H. Chiang , G. Varnavides , J. Xue , Y. Zhou , S. Park , R. Ding , J. Moon , G. Feng , P. Anikeeva , Nat. Nanotechnol. 2019, 14, 967. 31427746 10.1038/s41565-019-0521-z PMC6778020

[80] K. Y. Chan , M. J. Jang , B. B. Yoo , A. Greenbaum , N. Ravi , W.‐L. Wu , L. Sánchez‐Guardado , C. Lois , S. K. Mazmanian , B. E. Deverman , V. Gradinaru , Nat. Neurosci. 2017, 20, 1172. 28671695 10.1038/nn.4593 PMC5529245

[81] J. O. Szablowski , A. Lee‐Gosselin , B. Lue , D. Malounda , M. G. Shapiro , Nat. Biomed. Eng. 2018, 2, 475. 30948828 10.1038/s41551-018-0258-2

[82] E. Tunc‐Ozcan , C.‐Y. Peng , Y. Zhu , S. R. Dunlop , A. Contractor , J. A. Kessler , Nat. Commun. 2019, 10, 3768. 31434877 10.1038/s41467-019-11641-8 PMC6704083

[83] HERMES homepage, https://www.hermes-fet.eu/ (accessed: April 2021).

[84] E. Musk , bioRxiv 2019, 703801; 10.1101/703801.

